# Assistive technologies for pain management in people with amputation: a literature review

**DOI:** 10.1186/s40779-018-0151-z

**Published:** 2018-01-23

**Authors:** Kamiar Ghoseiri, Mostafa Allami, Mohammad Reza Soroush, Mohammad Yusuf Rastkhadiv

**Affiliations:** 1 Department of Orthotics and Prosthetics, School of Rehabilitation Sciences, Hamadan University of Medical Sciences, Hamadan, Iran; 2 Janbazan Medical and Engineering Research Center (JMERC), Farrokh Ave, Velenjak, Tehran, Iran; 3 Department of Occupational Therapy, School of Rehabilitation Sciences, Hamadan University of Medical Sciences, Hamadan, Iran

**Keywords:** Amputees, Amputation stumps, Self-help devices, Pain, Acute pain, Chronic pain, Pain management

## Abstract

The prevalence of limb amputation is increasing globally as a devastating experience that can physically and psychologically affect the lifestyle of a person. The residual limb pain and phantom limb pain are common disabling sequelae after amputation surgery. Assistive devices/technologies can be used to relieve pain in people with amputation. The existing assistive devices/technologies for pain management in people with amputation include electrical nerve block devices/technologies, TENS units, elastomeric pumps and catheters, residual limb covers, laser systems, myoelectric prostheses and virtual reality systems, etc. There is a great potential to design, fabricate, and manufacture some portable, wireless, smart, and thin devices/technologies to stimulate the spinal cord or peripheral nerves by electrical, thermal, mechanical, and pharmaceutical stimulus. Although some preliminary efforts have been done, more attention must be paid by researchers, clinicians, designers, engineers, and manufacturers to the post amputation pain and its treatment methods.

## Background

Limb amputation is a devastating experience that can physically and psychologically affect the lifestyle of a person [1]. Although there is no estimate of the global prevalence of limb amputation, national reports of some countries confirmed the increase in population of people with amputation [2, 3]. In most patients, amputation can cause two different kinds of pain, the phantom limb pain and the residual limb pain. Debilitating pain is associated with the burden of extra costs of treatment and the lost productivity of patients [4, 5]. Pain can affect the quality of life, outlook, personality, and relations of people with amputation. In addition, it can impede their rehabilitation and prosthesis use [6–9]. Kooijman et al. [10] in an epidemiological study, determined that the prevalence of phantom limb pain and residual limb pain is 51% and 47%, respectively.

## Category of pain

### Phantom limb pain

The majority of patients after a partial or complete amputation of a limb may feel that the amputated part of the body is still present and suffer from pain [4, 11–13]. In spite of the ample literature on phantom limb pain, there is no consensus on the exact mechanism of such a feeling. In the literature, phantom limb pain has been attributed to genetic background, memories, neuromas (the painful end branches of a cut nerve), peripheral/spinal dysfunction, supraspinal and central plasticity, and cortical re-mapping [14, 15]. Moreover, some physical, psychological, and weather-induced factors can increase the risk of phantom limb pain. Therefore, the existence of pre-amputation pain, the referral pain from the contralateral intact limb, neck or back, the emotional triggers such as stress, depression or thinking about the amputation, and temperature fluctuations all can trigger phantom limb pain [15]. Phantom limb pain in 50% of cases is an intermittent and episodic pain, which may range from hours, days, weeks, years, to decades [16]. Sherman et al. [16] found that 78% of their participant amputees had complaints of phantom limb pain. Moreover, the prevalence of phantom limb pain is higher in women and in those with upper extremity amputation [17].

### Residual limb pain

Residual limb pain can be described as the pain derived from physical damage to body tissues, especially at its distal end, during amputation surgery [13]. A main source of the residual limb pain is skin dermatosis, which is so prevalent in amputees with a range of 34% to 74% [18, 19]. Some common skin dermatoses include mechanically-induced problems, allergic reactions, and fungal infections [20]. Similar to phantom limb pain, the prevalence of residual limb pain is high. Yang et al. [21] reported the existence of residual limb pain in 61.5% of their 247 participant amputees.

## Pain management after amputation surgery

Pain management after amputation surgery can be classified into three categories, which include medical, non-medical, and surgical treatments. Surgical treatment is an invasive method that is usually considered as the last choice. Cordotomy, root lesion, targeted nerve implantation, and targeted muscle reinnervation are common surgical procedures to prevent or decrease residual limb pain and phantom limb pain [22–25]. The longevity of pain relief after surgical treatment is not high and usually the neuroma will grow again after surgery [15, 26, 27]. In spite of some drawbacks, the medication therapy has a great popularity. Anti-depression, muscle relaxation, analgesic, and opioid drugs are common examples of medications that are prescribed in spite of their side-effects [28, 29]. Contrary to great drawbacks of medication therapy and surgery, non-medical treatments have shown some promising results.

The most common non-medical treatment is using soft or rigid dressing over the residual limb to control pain and edema, and prevent joint contracture [30]. Some other non-medical treatments include nerve block, Botox injection, exercise therapy, massage, heat/cold pack, vibration and electroshock therapy, transcutaneous electrical nerve stimulation (TENS), acupuncture, psychological and behavioral treatments such as hypnosis and biofeedback (e.g. virtual reality methods such as mirror box therapy) [4, 28]. Although there are many disagreements about the beneficiary of non-medical treatments, they are more acceptable due to their fewer drawbacks. However, it is worth mentioning that some non-medical treatments such as nerve block may need some minor surgeries to place an assistive device/technology under the skin.

The assistive technology industrial association has defined assistive technology as any item, piece of equipment, software or product that can be used for increasing, maintaining, or improving the functional capabilities of individuals with disabilities [31]. The present review aimed to explore the literature to find existing assistive devices/technologies for pain management in people with amputation. Furthermore, the results of present review can provide an insight on current advances and limitations in pain management after amputation [32], which consequently, can promote peers to focus further on resolving the problem in future.

## Available assistive devices/technologies

### Electrical nerve block devices/technologies

**Fig. 1:**
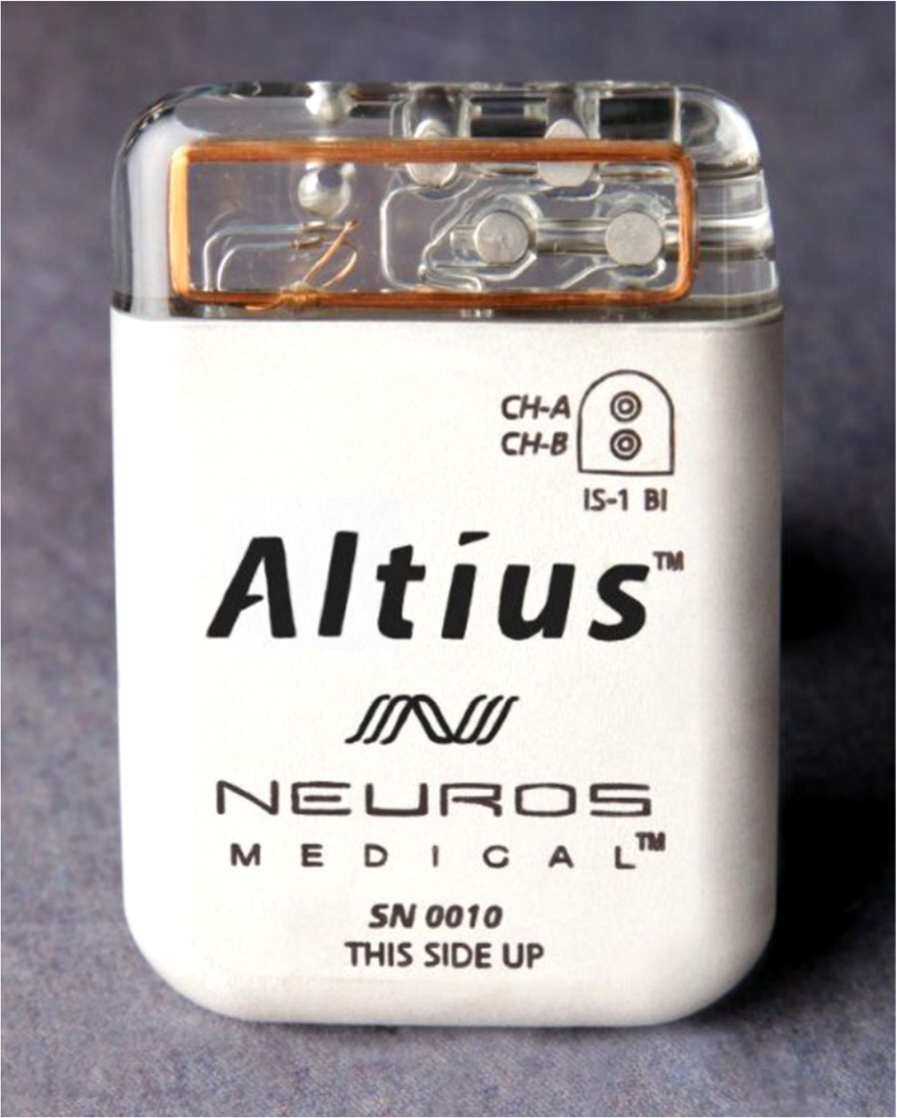
Electrical nerve block device/technology [https://www.neurosmedical.com/about/the-altius-system/]

Electrical nerve block can be used as an assistive device to alleviate pain in people with amputation [33]. In this regard, the Neuros Medical Inc. (Cleveland, OH, USA) has introduced a commercially available assistive device/technology to alleviate pain based on electrical nerve block. It is an electrical nerve blocker that applies high frequency sinusoidal waveforms of 10 kHz and up to 10 V proximal to the neuroma at a peripheral nerve (Fig. 1). This device consists of electrode, external or internal waveform generator, power supply, data logger, and an external remote controller to start pain relief when it is needed. The main problem of this assistive device is the need for surgery to implant the electrode around the nerve and its generator in a subcutaneous pocket in the abdominal region just below the rib cage. In spite of promising primary results of using this device to relieve pain in some amputees, more research is warranted to confirm its long time effects in a large group of amputees [33].

Rauck et al. [34] introduced another assistive device/technology for pain relief after amputation. The prototype of their device consisted of a fine-wire lead, an electrical stimulator, and a DC power supply. This prototype transferred electrical stimulations with a frequency of 50-100 Hz at a specific distance to the major peripheral nerves, i.e. femoral nerve or sciatic nerve, to produce paresthesia distal to the stimulation site. Their future plan was to develop the prototype as a smart patch nerve stimulator with skin-mounted stimulator and percutaneous lead after its safety and effectiveness evaluations.

### TENS units

**Fig. 2:**
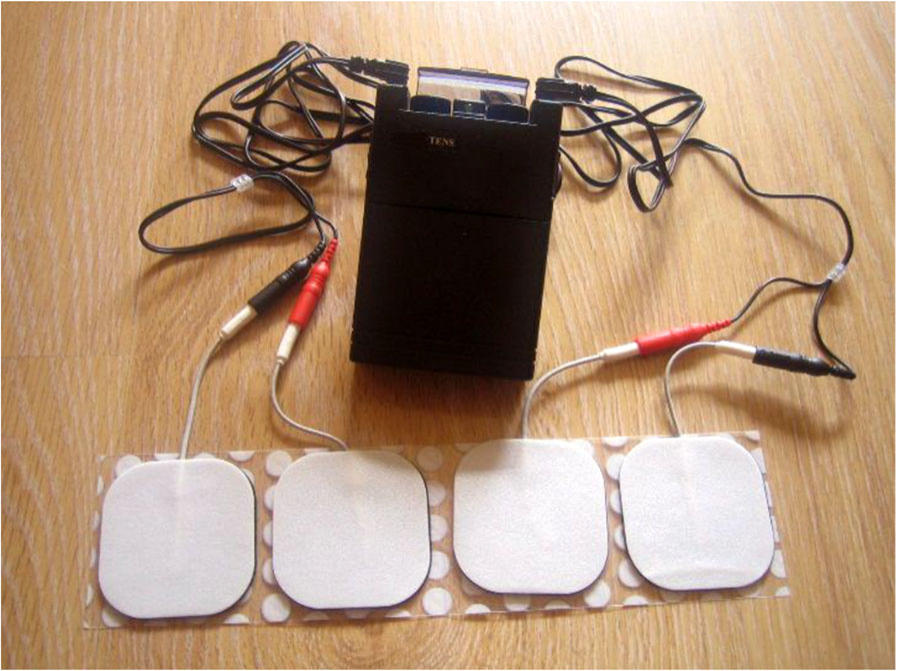
The transcutaneous electrical nerve stimulation unit [https://en.wikipedia.org/wiki/Transcutaneous_electrical_nerve_stimulation]

A TENS unit can be used as an assistive device/technology for pain relief after amputation (Fig. 2). Giuffrida et al. [35] used TENS on the contralateral (i.e. the healthy) limb of 2 participant amputees to evaluate its effect on pain relief. These authors confirmed the effectiveness of TENS in phantom limb pain relief. The effectiveness of TENS can be attributed to inhibiting the second order nociceptive neurons (analgesic effect), increasing blood flows, and reducing muscle spasms [36].

### Elastomeric pumps and catheters

**Fig. 3:**
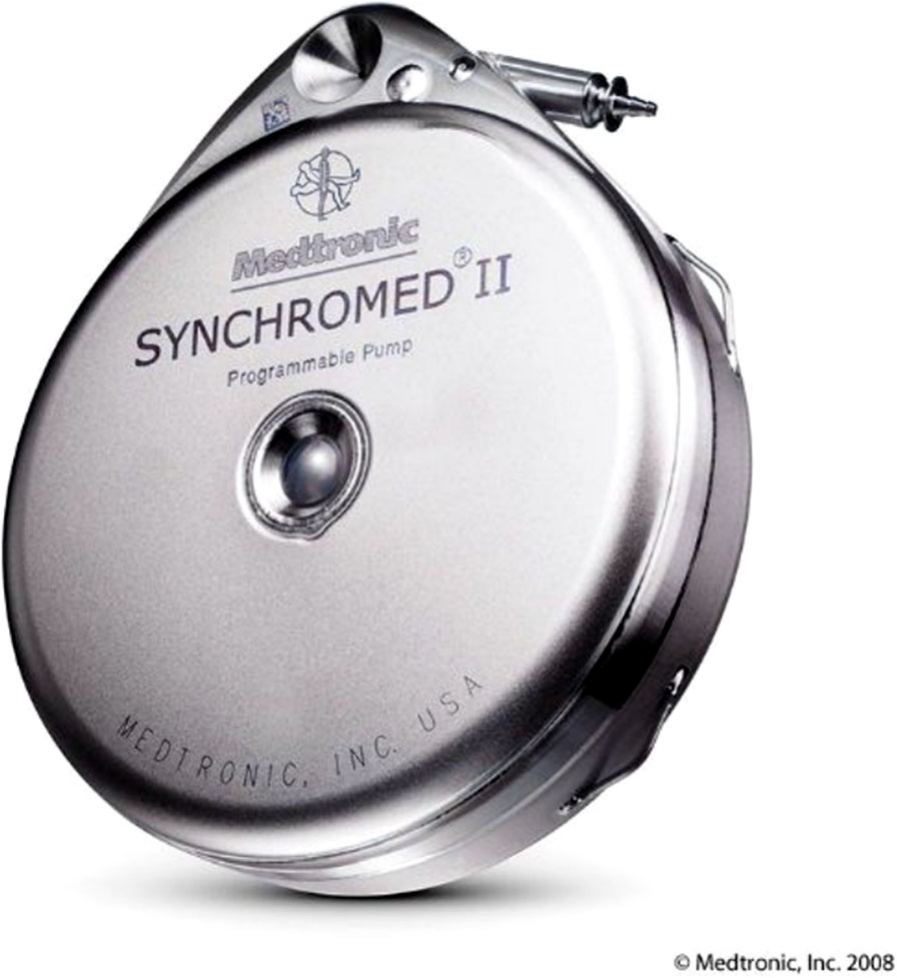
Elastomeric pumps and catheters [https://www.medtronic.com/us-en/healthcare-professionals/products/neurological/drug-infusion-systems.html]

Elastomeric pump and catheter is an assistive device/technology that can infuse local anesthesia for pain relief in people with amputation. The SynchroMed II infusion pump (Medtronics Inc., USA) is a commercially available assistive device/technology that produces analgesic effects by infusion of anesthesia (Fig. 3) [37]. This assistive device/technology is totally or partially implantable and can inject drugs to the subarachnoid or epidural space by activating a programmable or manual pump [38]. Therefore, the need for surgery, high cost, and high risk of infection are main problems associated with this assistive device/technology [38]. However, the results of a systematic review with meta-analysis showed that application of perineural local anaesthetic catheters alongside the transected sciatic nerve (for transfemoral amputations) or tibial nerve (for transtibial amputations) were possibly effective in pain relief [39]. Although they can approximately halve the postoperative opioid consumption, further investigation is warranted to determine their immediate pain relief after amputation [39].

### Residual limb covers

**Fig. 4:**
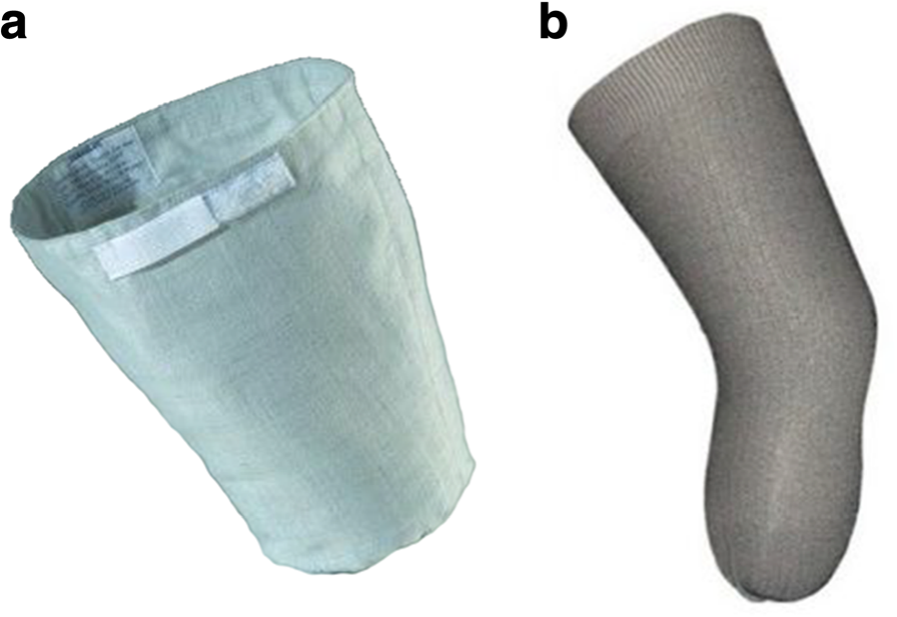
The Farabloc (**a**) and Medipro Relax (**b**) liners

Some specially designed and manufactured residual limb covers such as Farabloc and Medipro Liner Relax are applicable as assistive device/technology for pain relief in people with amputation (Fig. 4). The Farabloc was introduced by a Canadian company in 1993, as a special garment that comprised of a series of ultrathin steel threads woven into linen fabrics [40]. The Farabloc has the capability to prevent exposure of nerve endings of the residual limb to external electric and magnetic fields [40]. There are some controversial reports regarding the effectiveness of Farabloc. In a randomized controlled study, it was shown that Farabloc had no significant effect in reducing phantom limb pain after 12 weeks of use [41]. Zhang et al. [42] confirmed that the Farabloc was effective in alleviating delayed muscle soreness and pain in non-amputee people. Although satisfactory pain relief was reported by some amputees in the short-term use of Farabloc, further investigation is warranted to determine its effectiveness in the long-term, e.g. a year after amputation, use [40]. The Medipro Relax Liner was introduced in 2006 by a German-based company as an electromagnetically shielding liner with woven metals to cover the residual limb. This idea was derived from anecdotal experience of some people with amputation who wrapped their residual limb with aluminum foil to decrease phantom limb pain [43]. This liner has an electrical direct current with a resistance of 20 to 200 Ohms that flows from proximal to distal through an electromagnetic shield cover. Although it has been claimed that this electromagnetic shield significantly reduces the intensity of phantom limb pain, the mechanism of its action is unclear. Some possible mechanisms that have been suggested includes reduction of ectopic neuroma activity, shielding from electromagnetic impulses of weather, and analgesic effects due to changes in the electromagnetic field [43]. However, further research is required to confirm the effectiveness of this textile based liner and its underlying mechanism of action.

### Laser systems

Auricular acupuncture with laser might be another treatment method to alleviate post amputation pain [44]. Jacobs et al. [44] reported satisfactory pain relief using laser system in an amputee with phantom limb pain. However, the design of their study was not strong enough to confirm the effectiveness of this treatment method.

### Myoelectric prostheses

Myoelectric prostheses when equipped with some biofeedbacks are potentially assistive devices/technologies that can alleviate phantom limb pain [45]. However, further research is required to confirm their effectiveness.

### Virtual reality systems

**Fig. 5:**
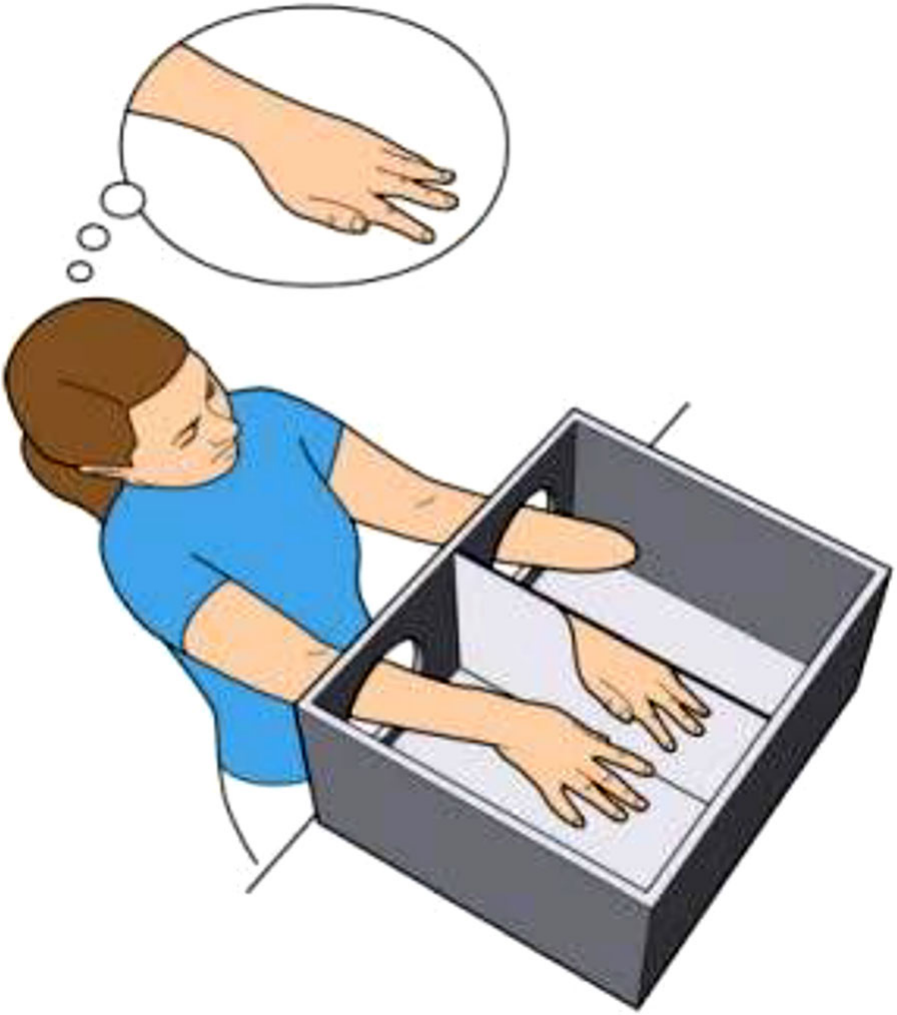
The virtual reality systems (mirror box)

The virtual reality is the last assistive device/technology that was found in this literature review [46]. It can be used separately or in conjunction with a biofeedback system to relieve pain in people with amputation. The motion capture technology and the brain computer interface are common biofeedback systems that can be connected to virtual reality [46–48]. The simplest type of a virtual reality device/technology is a rectangular box, i.e. the mirror box, without roof and front surfaces that has been divided in its middle by a vertical mirror (Fig. 5). The amputee sits in front of the box, places the intact limb on one side of the box and looks into the mirror. Therefore, it seems that the amputated limb revived and can be moved simultaneously with movements of the intact limb [49, 50]. This visual feedback causes the illusion in amputee and provides a bodily integrity sense that consequently can lead to cortical somatosensory reorganization. This process has been proved by MRI studies and reported as a satisfactory treatment for phantom limb pain [51]. However, further studies are required to confirm the effectiveness of virtual reality systems in alleviating phantom limb pain [52].

## Conclusions

The present review clearly showed that the majority of the available assistive devices/technologies could relieve pain conservatively. However, some of them are working by infusing a medication and some needs surgery to place some components inside body. Therefore, there is no distinct border among the 3 classified pain treatment methods, i.e. medical, non-medical, and surgical treatments, when assistive devices/technologies are applied in people with amputation. However, due to great drawbacks of medical and surgical treatments, future designs of assistive devices/technologies can focus more on non-medical treatments. There is a great potential to design, fabricate, and manufacture some portable, wireless, smart, and thin assistive devices/technologies to stimulate the spinal cord or peripheral nerves by electrical, thermal, mechanical, and pharmaceutical stimulus. Although some preliminary efforts have been done in this regard, there are few numbers of commercially available assistive devices/technologies for pain management in people with amputation [53–58].

The overall pain relief can’t easily be compared among assistive devices/technologies in this review due to different study designs, interventions, and characteristics of participants. Long-term randomized clinical trials are required to evaluate the effectiveness of available assistive devices/technologies. Considering the increasing population of people with amputation, high demand to assistive devices/technologies for pain management can be expected. Finally, more attention must be paid by researchers, clinicians, designers, engineers, and manufacturers to the post amputation pain and its treatment methods.

## Abbreviations

JMERC: Janbazan Medical and Engineering Research Center
TENS: Transcutaneous electrical nerve stimulation
